# Vaccinal Eruptions

**Published:** 1909-08-21

**Authors:** 


### VACCINAL ERUPTIONS.

There are several eruptions, such, for example, as eczema and Lichen urticatus, which are often wrongly put down by parents to the effects of vaccination. There are, however, certain forms of erythematous eruption which may be correctly ascribed to this cause; but they are not of common occurrence, and they are for the most part transitory and harmless. They may be regarded as analogous to similar eruptions which may occur as the result of the administration of certain drugs, or of serum or vaccines, or from absorption of food poisons. They may be scarlatiniform in type, morbilliform, roseolous, urticarial, or mainly limited to the limbs and simulating erythema multiforme. The erythematous eruption is often more intense on the limb about the vaccination sores, and thus may suggest erysipelas; but the presence of a less intense eruption on the trunk and limbs negatives this diagnosis. These eruptions usually appear while the vaccine pustules are at their acme, and subside in the course of a few days. There is a second group of eruptions which may complicate vaccination, all of which are to be avoided if proper precautions are taken. The infection of impetigo, of erysipelas, or even of syphilis might be introduced at the time of inoculation, but with calf-lymph and with antiseptic precautions these complications may be said not to occur at the present time. After the vaccination pustules have developed, there is again opportunity for infection with pus-organisms, and impetigo or erysipelas do sometimes develop, though usually only among the very poor. An accident which sometimes happens, though rarely, is re-inoculation of vaccine at a different site. This is possible up to the ninth or tenth day after vaccination. Instances of reinoculation have been recorded on the lesions of eczema, of varicella, of herpes zoster, and of impetigo. Accidental inoculation of a parent or nurse, often on the face, is not very uncommon. Possibly some of the cases of so-called generalised vaccinia may have been instances of re-inoculation.

